# Reply: chemotherapy for invasive papillary carcinoma: survival benefit or patient selection?

**DOI:** 10.1093/oncolo/oyag288

**Published:** 2026-08-03

**Authors:** Yi-Zi Zheng, Zhuo-Zhao Zhan, Ting-Ting Wu, Jia-Qi Ying, Ai-Na Zheng

**Affiliations:** Department of Breast Surgery, Zhejiang Key Laboratory of Intelligent Cancer Biomarker Discovery and Translation, The First Affiliated Hospital of Wenzhou Medical University, Wenzhou, Zhejiang, 325000, China; Department of Thyroid and Breast Surgery, the First Affiliated Hospital of Shenzhen University, Shenzhen Second People’s Hospital, Shenzhen University, Shenzhen, Guangdong, 518035, China; Department of Mathematics and Computer Science, Eindhoven University of Technology, 5612 AZ, the Netherland; Department of Breast Surgery, Zhejiang Key Laboratory of Intelligent Cancer Biomarker Discovery and Translation, The First Affiliated Hospital of Wenzhou Medical University, Wenzhou, Zhejiang, 325000, China; Department of Breast Surgery, Zhejiang Key Laboratory of Intelligent Cancer Biomarker Discovery and Translation, The First Affiliated Hospital of Wenzhou Medical University, Wenzhou, Zhejiang, 325000, China; The Operating Theater, The First Affiliated Hospital of Wenzhou Medical University, Wenzhou, Zhejiang, 325000, China

Dear editor,

In our original population-based SEER study, we analyzed 1892 patients with invasive papillary carcinoma (IPC) of the breast diagnosed between 2003 and 2021 and found that adjuvant chemotherapy was associated with improved overall survival (OS) in patients with lymph node-negative IPC tumors ≥2.0 cm after propensity score matching (PSM).^1^ We thank Dr. Ismayilov and Dr. Oguz for their insightful comments regarding our study on adjuvant chemotherapy for IPC. Their letter raises important considerations concerning the interpretation of survival data and its clinical applicability. We would like to address these points to provide further context for our findings.

First, we agree that residual confounding from unmeasured variables such as comorbidities and performance status cannot be fully excluded in retrospective SEER-based studies. To minimize treatment selection bias, we performed PSM using 14 clinically relevant variables. Importantly, these variables were selected not only because they are established prognostic clinicopathologic factors, but also because they are closely associated with real-world treatment allocation and the likelihood of receiving chemotherapy. After matching, no demographic variables with significant differences were observed. Furthermore, the significant overall survival benefit of chemotherapy persisted after PSM, despite much more balanced patient characteristics between groups (see figure 3A and C from the original article^1^). This finding does not support the hypothesis that the benefit was driven primarily by a healthier chemotherapy group. Regarding the discordance between OS and breast cancer-specific survival (BCSS), we respectfully note that IPC is a relatively indolent subtype with a limited number of breast cancer-specific events, particularly after subgroup stratification and matching. Therefore, the BCSS analysis may have been underpowered to detect a statistically significant difference despite the observed OS benefit. Similar OS-BCSS discrepancies are not uncommon in retrospective studies of rare, favorable-prognosis breast cancer subtypes.^2^

**Figure 1.**
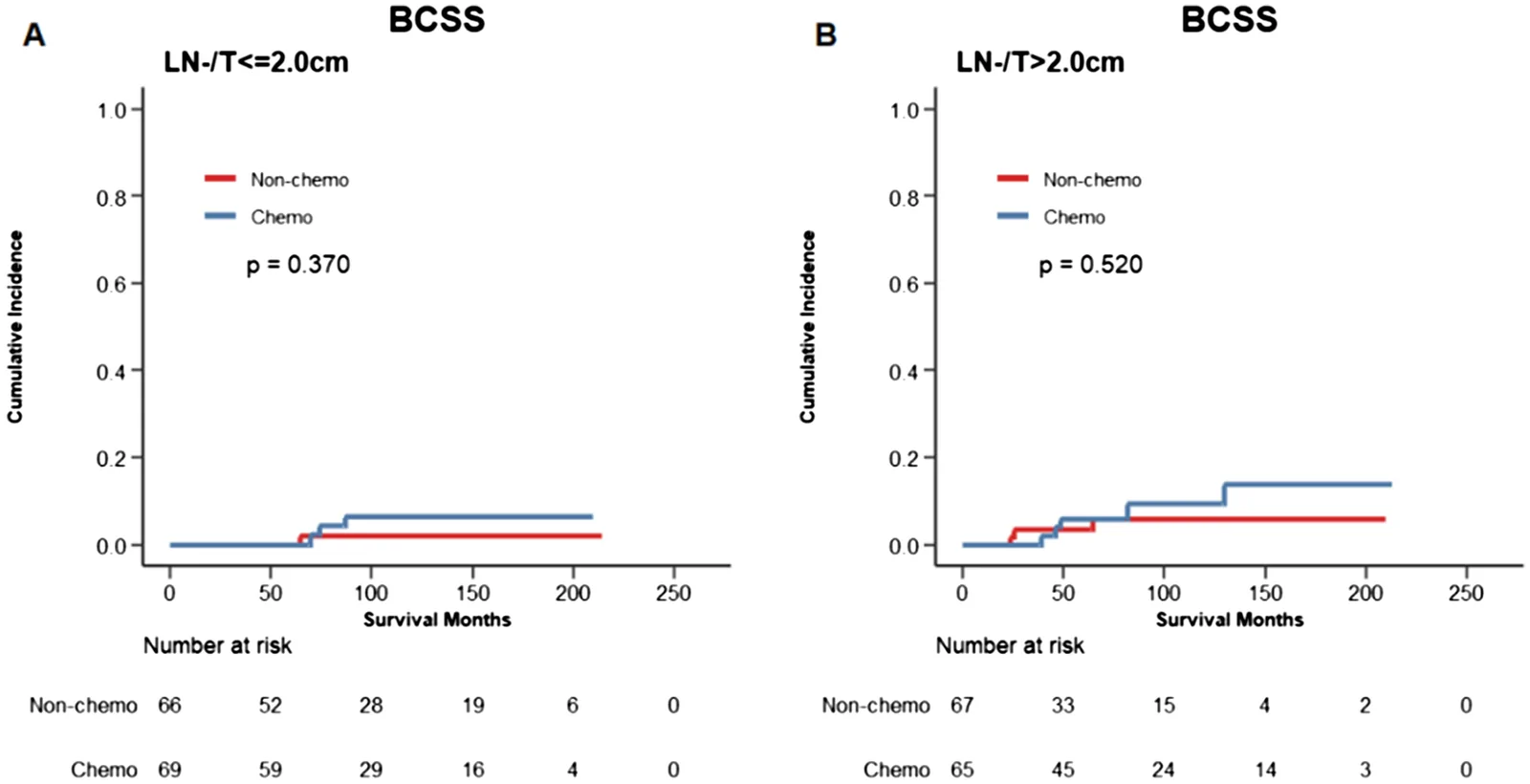
(A) Breast cancer-specific survival (BCSS) using the Fine–Gray competing risks model in lymph node-negative patients with tumors <2.0 cm after propensity score matching. (B) BCSS using the Fine–Gray competing risks model in lymph node-negative patients with tumors ≥2.0 cm after propensity score matching.

If breast cancer-specific mortality was underestimated in the non-chemotherapy group—to a greater degree than in the chemotherapy group, due to higher comorbidity, competing causes of death, and greater frailty—one would expect the survival difference between the groups to widen after PSM. As shown in Figures 3 and 4, we observed no such widening. The reviewers suggested that model choice could confound this result and recommended the Fine–Gray model instead of the cause-specific Cox model (which censors competing events). We therefore repeated the analyses using the Fine–Gray model and obtained results consistent with the cause-specific Cox approach (see Figure 1A and B below). While the Fine–Gray model excels at absolute risk prediction, its subdistribution hazard can be difficult to interpret clinically because it retains individuals who died from other causes in the at-risk denominator. For our study objective, the cause-specific hazard is more appropriate: it directly answers the question, “Does chemotherapy reduce the rate of death specifically from breast cancer?” and thus provides a straightforward evaluation of the treatment’s cytotoxic benefit on the malignancy itself in a clinical setting.

Second, we fully agree that genomic profiling, including the 21-gene recurrence score validated in the TAILORx trial, represents the contemporary standard for guiding adjuvant chemotherapy in hormone receptor-positive, HER2-negative, node-negative breast cancer. However, IPC is an exceptionally rare histological subtype, accounting for only approximately 0.5%-2% of invasive breast cancers. Consequently, IPC was either absent or represented only a very small minority in large prospective genomic assay trials such as TAILORx, and the evidence supporting genomic-guided treatment decisions in IPC remains substantially less robust than that for conventional invasive ductal carcinoma.

Additionally, real-world accessibility of genomic testing varies widely due to financial, insurance, geographic, and socioeconomic barriers. Our study does not aim to replace genomic-guided decision-making but provides supplementary evidence based on readily available clinicopathological parameters (particularly tumor size and nodal status) for settings where genomic testing is unavailable, inaccessible, or declined.

We believe these clarifications strengthen the interpretation of our findings.
